# Notes of Travel

**Published:** 1854-01

**Authors:** 


### SELECTIONS.

From the Peninsular Journal of Medicine.

### *Notes of Travel.* By "X."

He that wants to know how a thing is, must go and see it. If you want to know how deep a stream is, there is no way so good as to go and measure it. So lately, upon a time, while jogging about in Michigan and the adjoining states, it occured to me to take the demensions by actual statistics, of that Jordan of Quackery which Behemoth Bullhead "trusteth that he can draw up in his *Corporal* mouth:" for this came Jordan has roared and foamed and sputtered, until many people suppose it to be exceeding deep and mighty, and if the Corporal and the Q. K. army purpose to precipitate themselves into any rash undertaking, we must hold on to their over-valiant coat tails, lest they be "devoured in battle." Moved by these humane considerations I took statistical notes of such places as I visited, or could obtain reliable information about. The brief result of my examination, is, that there is more foam than fact about the quack clans, and any body will do Corporal Bullhead a service, who will get enough of them together into one phalanx, to make it worth his while to charge them. The results of my inquiries may be found in the subjoined table. From various causes the numbers may contain some errors. Changesare frequent, and though but a few weeks have elapsed, removals have by this time, transferred some practitioners, and many quacks, to other locations; then there were men who had partly retired from practice, and it was noteasy to say whether they were still to be classed as acting physicians or not; and there were quacks, who held themselves up to practice anything and accommodated themselves to the whims of their patrons, so that it was a matter of difficulty, to know whether to call them Eclectics, Homœopaths, Botanists, or what. On account of these various sources of ambiguity, it was not possible to class them with perfect precision, but as a whole the table must be very nearly accurate. I have placed Homœopaths and Eclectics by themselves, because they boast the loudest of their numerical strength and supposed increase of power. All other quacks I put together under the head "Miscellaneous:"

### TABLE.

| *Places.* | *Regulars.* | *Homœopathists.* | *Eclectics.* | *Miscellaneous.* |
|---|---|---|---|---|
| Detroit, | 40 | 8 | | 6 |
| Dearborn, | 2 | | | |
| Ypsilanti, | 6 | 1 | | 1 |
| Ann Arbor, | 7 | 3 | | |
| Dexter, | 3 | 2 | | |
| Jackson, | 9 | 2 | | 1 |
| Albion, | 8 | | 1 | 1 |
| Marshall, | 7 | 1 | 1 | 1 |
| Galesburg, | 2 | | | |
| Battle Creek, | 8 | 1 | | 3 |
| Kalamazoo, | 10 | 2 | | |
| Paw Paw, | 3 | 2 | | |
| Niles, | 6 | | 1 | |
| Michigan City, | 5 | | | 1 |
| Laporte, Ia., | 6 | 1 | 2 | |
| S. Bend, Ia., | 7 | 1 | 4 | |
| Mishawaka; In., | 4 | | 1 | |
| White Pigeon, | 2 | | | |
| Coldwater, | 9 | | | 3 |
| Jonesville, | 3 | 1 | | |
| Hillsdale, | 7 | | | |
| Adrian, | 5 | 1 | 2 | 2 |
| Toledo, O., | 12 | 1 | | 3 |
| Monroe, | 8 | | | |
| Eaton Rapids, | 3 | 1 | 1 | |
| Lansing, | 3 | | | 1 |
| Portland, | 2 | | | |
| Sebewa, | 1 | | | 1 |
| Lyons, | 3 | 1 | | |
| Ionia. | 4 | | 1 | |
| Lowell, | 1 | | | |
| Grand Rapids, | 7 | 1 | | 1 |
| Middleville, | 1 | | | |
| Hastings, | 4 | | | |
| Schoolcraft, | 2 | | | 1 |
| Lawrence, | 1 | | | 1 |
| Breedsville, | 1 | | | |
| Pontiac, | 7 | 1 | | 1 |
| Mt. Clemens, | 5 | | | 1 |
| Utica, | 2 | | | 2 |
| Washington, | 2 | | | 1 |
| Rochester, | 3 | | | |
| Romeo, | 3 | | | |
| Royal Oak, | 3 | | | |

| *Places.* | *Regulars.* | *Homœopathists.* | *Eclectics.* | *Miscellaneous.* |
|---|---|---|---|---|
| Birmingham, | 3 | | | |
| Constantine, | 5 | | | 1 |
| Mottville, | 2 | | | |
| Centreville, | 3 | | | |
| Union City, | 4 | 1 | 1 | |
| Total, | 255 | 32 | 15 | 36 |

Total of all Practitioners 338.

These places were taken at random as specimens of the territory in which they lie, and probably give a pretty fair view of the relative strength of Quackery in this region. I think the region as a whole, whould not differ very widely from the percentages of the samples here given.

Of the entire number of practitioners, it will bo seen by the table that about one in four is a Quack, about one in ten is a Homœopath, and one in tweuty-two is an Eclectic.

The local distribution is also a curious point to be observed. Take the line of the Central Railroad from Detroit to Paw Paw, and it may be called the Homœopathic region, they being one-seventh of all the practitioners; while in the remainder they number only one in nineteen.—I commend these facts to the consideration of those who hear the loud boasts of his Homœopathic district, where little pill men talk and roar, as though this little patch of pillets were just on the verge of being a majority of the state. So far as I could disoover, Homœopathic seemed to be about stationery in actual numbers, and declining, if compared with the increase of population. I found a considerable number of large places where it had died out entirely.

On the Southern Railroad there is a similar patch of Eclectics. If you take from the table the places from Laporte, (Ind.,) to Adrian, the Eclectics will be found to number about one-seventh of the practitioners, and the Homœopaths one-fifteenth.

The remaining quacks consists of Botanics, Hydropaths, Root Doctors, etc., etc., all of which I have classed under the head "Miscellaneous." I found a small water-cure establishment at Coldwater, but no one seemed to know much about it. I was informed that it had eight or ten patients in it. A case of death by cold water took place there just before I arrived. A Thomsonian doctor, who had become partly converted to Hydropathic, was taken sick. He deemed that his valuable life required the potent virtues of two systems of quackery to save it, so he forthwith swallowed a goodly prescription of red peper and whiskey, and then sent for the Water-Cure doctor to come and treat him. The Hydropath put him in a wet sheet, ordered him to drink very freely of cold water, and left him for the night. Not being very desirous to render to the bar of justice just then his soul's final account, he followed the directions vigorously. The nurse solemnly avers that she brought him two buckets of water in the night, and that he drank it all. How this is, I do not know, but one thing is certain, in the morning he "kicked the bucket," and went to settle his accounts with those of his patients, that had gone from his treatment to the other world before him. I saw in the same vicinity, a young man with a hopeless disease of the heart, brought on by cardiac rheumatism, transferred to that organ from other parts by cold water applications.

Those who watch the signs of the times in our profession, may, by help of the above table, mark one thing. The success of quack systems, is limited, localised, and interrupted by interference with each other: there is competition in the market, and *pathy* stocks must fall. There is a law of their rise and decline, which cannot be evaded. Once, patent medicines were all the rage, and enormous fortunes realized for a time, by those who got them early enough into the market to get a universal circulation. This started up thousands of other nostrums-inventors from every corner of the land; each anxious to make a huge fortune by puffing his remedy into use. But this could not last. Secret remedies were no longer wonderful when every newspaper advertised fifty. In such a crowd, no one could be distinguished, so the whole fell into disrepute, and although abundance of nostrums are sold, their day of glory is over. The same principles now hold in regard to *pathies*. It is the day of *system of remedies* now, the next natural step after the day of *single* remedies. But these are already too many to gain the highest glory,—they do not any of them go up with such eclat as did Brandreth's Pills,—and the machinery of propulsion for keeping them in motion is necessarily more ponderous and slow, but the principle is the same. If one *pathy* shines, a dozen more will spring up to share its money-making, and though they will live longer than patent medicines, their end is the same,—by multiplication they destroy each other He that at this day invents a new quack system of practice, is a scoundrel it is true, but he is doing the world service. These are some of my hasty thoughts after surveying the field. I have dwelt entirely in my statistics on *numerical* strength, because that can be estimated in numbers, but the impression that remains upon my mind, of the intellectual and moral power of the profession of this region, as a body, cannot be thus expressed. There is in it a peculiar aspect of cool and calm strength, overlying an amount of science and attainment, and backed up by solid elements of power and influence, that make vapory nonsense of the clamors of our enemies. I can but be rejoiced at the present even, and exult for the coming future.

